# Investigation on the Power Factor of Skutterudite Sm*_y_*(Fe*_x_*Ni_1−*x*_)_4_Sb_12_ Thin Films: Effects of Deposition and Annealing Temperature

**DOI:** 10.3390/ma14195773

**Published:** 2021-10-02

**Authors:** Giovanna Latronico, Paolo Mele, Cristina Artini, Pietro Manfrinetti, Sian Wei Pan, Yukihiro Kawamura, Chihiro Sekine, Saurabh Singh, Tsunehiro Takeuchi, Takahiro Baba, Cédric Bourgès, Takao Mori

**Affiliations:** 1 Shibaura Institute of Technology, Omiya Campus, 307 Fukasaku, Minuma-ku, Saitama City 337-8570, Saitama, Japan; pmele@shibaura-it.ac.jp; 2 Department of Chemistry and Industrial Chemistry, University of Genova, Via Dodecaneso 31, 16146 Genova, Italy; artini@chimica.unige.it (C.A.); chimfis@chimica.unige.it (P.M.); 3 Institute of Condensed Matter Chemistry and Technologies for Energy, National Research Council, CNR-ICMATE, Via De Marini 6, 16149 Genova, Italy; 4 Muroran Institute of Technology, 27-1 Mitsumoto-cho, Muroran 050-8585, Hokkaido, Japan; 21043057@mmm.muroran-it.ac.jp (S.W.P.); y_kawamura@mmm.muroran-it.ac.jp (Y.K.); sekine@mmm.muroran-it.ac.jp (C.S.); 5 Toyota Technological Institute, 2-12-1 Hisakata Tenpaku, Nagoya 468-8511, Aichi Perefecture, Japan; saurabhsingh@toyota-ti.ac.jp (S.S.); t_takeuchi@toyota-ti.ac.jp (T.T.); 6 International Center for Materials Nanoarchitectonics (WPI-MANA), National Institute for Materials Science (NIMS), Namiki 1-1, Tsukuba 305-0044, Ibaraki, Japan; baba.takahiro@nims.go.jp (T.B.); bourges.cedric@nims.go.jp (C.B.); MORI.Takao@nims.go.jp (T.M.)

**Keywords:** thermoelectricity, skutterudites, thin films, pulsed laser deposition, power factor, thermal conductivity

## Abstract

Filled skutterudites are currently studied as promising thermoelectric materials due to their high power factor and low thermal conductivity. The latter property, in particular, can be enhanced by adding scattering centers, such as the ones deriving from low dimensionality and the presence of interfaces. This work reports on the synthesis and characterization of thin films belonging to the Sm*_y_*(Fe*_x_*Ni_1−*x*_)_4_Sb_12_-filled skutterudite system. Films were deposited under vacuum conditions by the pulsed laser deposition (PLD) method on fused silica substrates, and the deposition temperature was varied. The effect of the annealing process was studied by subjecting a set of films to a thermal treatment for 1 h at 423 K. Electrical conductivity *σ* and Seebeck coefficient *S* were acquired by the four-probe method using a ZEM-3 apparatus performing cycles in the 348–523 K temperature range, recording both heating and cooling processes. Films deposited at room temperature required three cycles up to 523 K before being stabilized, thus revealing the importance of a proper annealing process in order to obtain reliable physical data. XRD analyses confirm the previous result, as only annealed films present a highly crystalline skutterudite not accompanied by extra phases. The power factor of annealed films is shown to be lower than in the corresponding bulk samples due to the lower Seebeck coefficients occurring in films. Room temperature thermal conductivity, on the contrary, shows values comparable to the ones of doubly doped bulk samples, thus highlighting the positive effect of interfaces on the introduction of scattering centers, and therefore on the reduction of thermal conductivity.

## 1. Introduction

The increasing global energy demand, together with the very real greenhouse emissions and global warming issues, is becoming increasingly alarming, leading scientists to search for new sustainable energy sources and energy-saving routes. Undoubtedly, in order to reduce the carbon footprint, several alternatives should be explored, such as solar [1] and wind [2] power, as well as fuel cell [3,4] technologies. In this scenario, thermoelectricity is a promising and attractive property of materials which could play a notable role in the future global requirement of energy due to its dual function in power generation and refrigeration. Thermoelectric generators (TEGs) can be employed for a wide spectrum of purposes, for example as radioisotope heat sources for space applications, or as power supplies in remote areas, or in the automotive field [5,6]. Especially for the thin films discussed in this paper, the applicative possibilities as thermoelectric energy harvesting to power Internet of Things (IoT) sensors and devices are important [7,8,9,10].

The thermoelectric (TE) materials’ performance is evaluated through the dimensionless figure of merit *ZT*:

(1) $$ZT=\frac{\sigma S^{2}}{k}$$

with σ being the electrical conductivity, S the Seebeck coefficient, T the absolute temperature and k the thermal conductivity. Furthermore, k consists of the sum of the electronic thermal conductivity $k_{e}$ and the lattice thermal conductivity $k_{L}$ [11]. In addition, $k_{e}$ and σ are firmly correlated to each other through the Wiedemann–Franz law:

(2) $$k_{e}/\;\sigma =L\;T$$

with *L* being the Lorentz number. The optimization of ZT points toward boosting power factor (*PF*), namely the $\sigma S^{2}$ product, and abating thermal conductivity. σ and S show an opposite behavior depending on doping and charge carrier concentration; since, as shown before, σ and $k_{e}$ are interdependent, individually adjusting these parameters is a very difficult task, which requires deep knowledge of the material band structure. The phonon contribution to thermal conductivity $k_{L}$, on the contrary, is essentially independent of σ and S, and the attempt to minimize its value by reducing the phonon mean free path is the most easily accessible phenomenological approach to the optimization of thermoelectric materials. This idea relies on the expression of $k_{L}$:

(3) $$k_{L}=\frac{1}{3}C_{v}vl$$

where $C_{v}$ is the specific heat at constant volume, v the sound velocity, and l the phonon mean free path. The most common technique consists of the introduction of scattering centers, for instance through density enhancement [12], porosity control [13], nanostructuring [14], mesostructuring [15] or precipitation of nano-sized secondary phases [16]. A further method, consisting of the introduction of substitutional and/or interstitial atoms, underlies the optimization of skutterudites.

Given all the described conditions, it has become clear that the best compromise, able to reconcile the maximization of σ and S and the minimization of $k_{L}$, can be found in semiconductors [17]. Among the many classes of materials currently studied, such as Heusler phases [18] and clathrates [19], filled skutterudites play a relevant role due to the possibility of obtaining *n*- and *p*-conducting materials within the same system just by changing the number of doping ions [20].

Skutterudites MX_3_ [21,22] (M ≡ transition metal, X ≡ pnictogen atom) crystallize in a body-centered cubic cell (Pearson symbol *cI*32, $Im\bar{3}$ space group, isotypic crystal: CoAs_3_) with two different atomic sites, namely the 8*c* (¼, ¼, ¼) and the 24*g* (0, *y*, *z*), occupied by M and X, respectively. MX_6_ strongly tilted corner-sharing octahedra, and an X_12_ icosahedral cage with its center in the 2*a* site located in (0, 0, 0), consequently form. Such a material is characterized by a value of k that is too high to be exploited for thermoelectric applications [23], but if a proper R atom (R ≡ rare earth or alkaline-earth element) enters the cavity, $k_{L}$ is significantly lowered thanks to the vibrational modes of the vibrating guest, which hinder the propagation of heat-carrying phonons [24], and ZT is consequently enhanced. In this respect, filled skutterudites RM_4_X_12_ follow the phonon-glass electron-crystal (PGEC) concept [11], stating that ideal thermoelectric materials should conduct heat like glass and electricity like a crystal, and thus have low thermal conductivity and high electrical conductivity. Some examples of high-quality outcomes are the *n*-type (Sr,Ba,Yb)Co_4_Sb_12_ with *ZT* ≈ 2.0 at 835 K [25] and the *p*-type DD_0.7_Fe_3_CoSb_12_ with *ZT* > 1.3 at 856 K [26]. As previously mentioned, in the majority of filled skutterudite systems, both *p*- and *n*-type compounds can be synthesized from the same parent compound by tuning the filling ratio of the R atom and the partial substitution of the M atom, thus creating a lot of different compounds, such as Fe/Ni- [27,28] and Fe/Co- [29,30] based ones. At the same time, the insertion of two different M atoms, similarly to the doping by two different rare earths [27] or the partial substitution of Sb by Sn [31] or Ge [32], is responsible for the creation of additional scattering centers, and thus for a further lowering of $k_{L}$.

The reasons behind the study of skutterudite thin films are therefore twofold: on one hand, a basic scientific motivation leads us to investigate the effect of low dimensionality and the presence of interfaces, which make thin films particularly interesting as a source of further phonon scattering [33,34]. On the other hand, from a technological point of view, it is desirable to produce flexible and robust TE devices, able to harvest heat from curved or irregularly shaped surfaces, overcoming the flat, bulky and fragile commercial TE devices. Nevertheless, in spite of the relevance of these issues, very few works are devoted to the deposition of skutterudite thin films and to their characterization, and almost all of them deal with CoSb_3_ [35,36] and CoSb_3_-deriving compounds [33,37,38].

This work reports on the deposition by pulsed laser deposition of thin films of two filled skutterudites belonging to the Sm*_y_*(Fe*_x_*Ni_1−*x*_)_4_Sb_12_ system. The effects on the structural and transport properties of the deposition and the annealing temperature were studied. The deposition at room temperature, followed by an annealing process at 423 K, was revealed to be essential for the obtainment of crystalline films and preferable to a high-temperature deposition. While power factor is lower in films than in the corresponding bulk samples due to the lower Seebeck coefficients, room temperature thermal conductivity exhibits values comparable to the ones of doubly doped bulk samples; this encouraging result suggests the positive effect of the presence of interfaces on the introduction of scattering centers, and therefore on the reduction of thermal conductivity. Starting from this point, our future works will be focused on achieving further reduction in thermal conductivity by introducing artificial nanoparticles.

## 2. Materials and Methods

### 2.1. Preparation of Porous Samples and Dense Targets

Two compositions of the Sm*_y_*(Fe*_x_*Ni_1−*x*_)_4_Sb_12_ filled skutterudite system were prepared by the conventional melting–quenching–annealing technique with nominal (*x* = 0.63; *y* = 0.20) and (*x* = 0.70 *y* = 0.40), being the former at the *n/p* crossover, and the latter *p*-conducting, respectively [39]. The Sm content was chosen based on the results described in [28]. Additionally, Sb was added in a slight excess compared to the stoichiometric quantity in order to compensate for the possible loss caused by its non-negligible vapour pressure (0.133 Pa at 873 K [40]). Small pieces of pure elements (Fe, Alfa-Aesar, 99.99 wt.%; Ni, (Alfa-Aesar, Kandel, Germany), 99.99 wt.%; Sm (NewMet, Waltham Abbey, UK); 99.9 wt.%; and Sb (Mateck, Jülich, Germany), 99.99 wt.% were weighted in the specified amounts and placed under vacuum in quartz tubes. The mixtures were then thermically treated at 1223 K for 1 h to ensure the homogenization of the liquid phase, and then hastily cooled in a water bath to improve microcrystallinity and facilitate the subsequent annealing process. As-cast samples were then annealed in vacuum at 873 K for 7 days in order to promote the formation of the desired phase, and subsequently ground in mortar operating within an Ar-filled glovebox in order to prevent oxidation.

The sample with *x* = 0.63 was densified by spark plasma sintering (SPS, home-made machine at the University of Pavia, Italy) at 773 K for 5 min under a pressure of 50 MPa. Discs’ diameters ranged between 1 and 1.5 cm, depending on the availability of the starting material. Targets of specimens with *x* = 0.70 were prepared by the open die pressing technique (ODP, at CNR-ICMATE in Lecco, Italy). Powders were encapsulated into an iron sleeve; the inner surface was covered with a layer of BN to prevent sticking and to facilitate the sample removal after the process. The specimen was preheated at 773 K for 3 min and pressed with its axis horizontally oriented between two heated plates of a hydraulic press. The dense sample is provided with a characteristic shape, giving the possibility to obtain two distinct targets. Samples are named Fe63 and Fe70, depending on Fe % amount with respect to the total (Fe + Ni) content.

### 2.2. Characterization of Porous Samples and Dense Targets

Morphology and composition of both porous and dense samples were studied by electron microscopy coupled to energy dispersive X-ray spectroscopy (SEM-EDS, Oxford Instruments, Abingdon, UK, model 7353 with Oxford-INCA software v. 4.07, Link Analytical – Oxford Instruments, Abingdon-on-Thames, UK, working distance: 15 mm, live time: 40 s); to this purpose small pieces were encapsulated in resin and micrographically polished prior to being analyzed. EDS analyses were performed on at least five points for each selected area. Microhardness of porous samples was measured by means of a VMHT microhardness tester (Leica, Wetzlar, Germany) provided with Vickers indenter. A test load of 50 g was applied with a dwell time of 15 s; 10 tests were performed on each sample.

Powders obtained from grinding bulk samples were analyzed through X-ray diffraction by a Bragg–Brentano powder diffractometer (Philips PW1050/81, Amsterdam, The Netherlands, Fe-filtered Co K_α_ radiation, power settings: 30 mA, 40 kV) making use of a zero-background sample holder in the 20°–110° angular range.

### 2.3. Deposition of Thin Films

Filled skutterudite thin films were grown by the pulsed laser deposition (PLD) technique using a Nd:YAG (266 nm, 10 Hz) laser (LOTIS TII, Minsk, Belarus). Squared silica pieces were chosen as substrates, undergoing firstly a cleaning process at 773 K for 2 h, and then being glued by means of silver paint on an Inconel plate which was then inserted into the PLD chamber. The laser was shot on the dense Fe63 and Fe70 targets with an energy density of about 4.2 J/cm^2^ for a deposition time of 60 min under high vacuum (10^−4^ Pa). Films were deposited both at room temperature and at 423 K, and a set of the former batch was subjected to an annealing process at 423 K for 1 h under a flux of argon gas (200 cm^3^/min). Films are named Fe63_RT, Fe70_RT, Fe63_423, Fe70_423, Fe63_ann and Fe70_ann according to the Fe amount, as previously elucidated, and to the process the sample was subjected to: RT (deposition at room temperature), 423 (deposition at 423 K), ann (annealed). In Table 1, an overview of the experimental conditions of film deposition is reported.

### 2.4. Characterization of Thin Films

Morphology and composition of thin films’ surfaces were studied by electron microscopy coupled to energy-dispersive X-ray spectroscopy (lower electron detector, LED-SEM, JEOL/JSM-7100F, Akishima, Japan); to this purpose, the surface of the films was coated with gold prior to being analyzed. EDS analyses were performed on at least five points for each selected area. The thermoelectric parameters, such as electrical conductivity (σ) and Seebeck coefficient (S), were concurrently measured by the four-probe method between 348 and 523 K using a ZEM-3 (ULVAC Advance-Riko, Chigasaki, Japan) apparatus under a partial He pressure to assure the thermal transport between the heater and the sample. The thickness of samples was evaluated by means of a Dektak 6M Stylus profilometer (Bruker, Billerica, MA, USA).

All samples were subjected to the X-ray diffraction analysis, both before and after the thermoelectrical characterization by a Bragg–Brentano powder diffractometer (Smart Lab3 Rigaku Corporation, Tokyo, Japan) using the Cu K_α_ radiation in the 10°–100° angular range with angular step 0.02° (power settings: 40 mA, 40 kV).

The picosecond time-domain thermoreflectance (TD-TR) technique using a customized focused thermal analysis system based on PicoTR (PicoTherm, Tsukuba, Japan) was utilized to measure thermal conductivity of the samples at room temperature in the cross-plane direction [41,42,43,44]. Details are reported in the section Appendix A.

## 3. Results and Discussion

### 3.1. Morphological, Compositional and Structural Properties of Porous and Dense Samples

**Figure 1:**
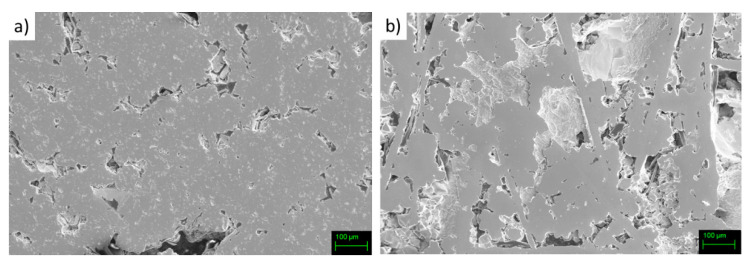
SE-SEM microphotographs of the polished surface of bulk samples (**a**) Fe63 and (**b**) Fe70.

The surface of bulk annealed samples appears highly porous, as depicted in Figure 1a,b, where microphotographs taken on both samples by secondary electrons (SE) are shown.

**Figure 2:**
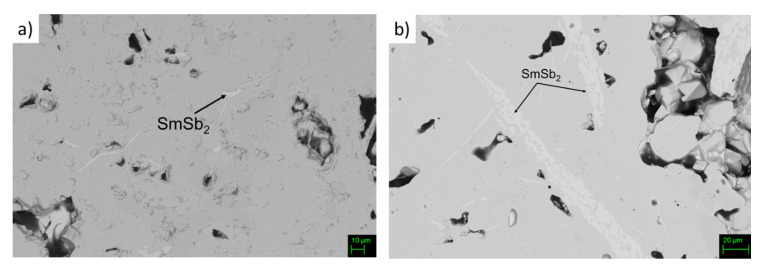
BS-SEM microphotograph of the polished surface of sample (**a**) Fe63 and (**b**) Fe70.

Backscattered (BS) images and EDS analyses suggest that the main phase is the desired skutterudite, with composition Sm_0.17_(Fe_0.60_Ni_0.40_)_3.75_Sb_12_ and Sm_0.38_(Fe_0.69_Ni_0.21_)_3.75_Sb_12_ for samples Fe63 and Fe70, respectively. Nevertheless, the presence of the additional phase SmSb_2_, which is commonly found in non-perfectly monophasic samples belonging to this system [45], can be observed too, as can be inferred from Figure 2.

**Figure 3:**
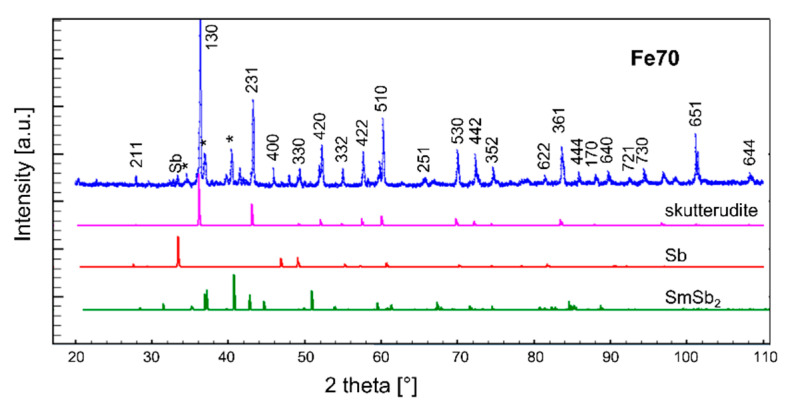
X-ray diffraction pattern of sample Fe70 and calculated diffractograms of skutterudite, Sb and SmSb_2_. In the experimental pattern, Miller indexes of the skutterudite phase are indicated, as well as the position of the main peaks of the additional phases Sb and SmSb_2_ (asterisks).

The presence of the aforementioned additional phase, as well as of a tiny amount of Sb, is confirmed by the results of X-ray acquisitions, as observable in Figure 3.

Microhardness was measured at several points of both phases on the Fe70 surface; results clearly show a clustering of data around two values, namely 462(23) and 373(14) HV, the former being associated to skutterudite and the latter to the SmSb_2_ extra phase. Such a value for skutterudite is in good agreement with the values of samples belonging to the (Sm,Gd)*_y_*(Fe*_x_*Ni_1−*x*_)_4_Sb_12_ system [27].

**Figure 4:**
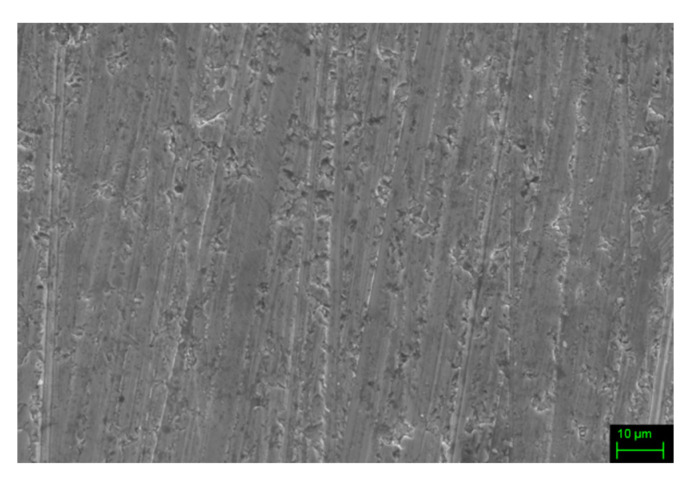
SE-SEM microphotograph taken on dense Fe70 sample.

After the densification process, both compositions show a significant density enhancement, as clearly depicted in Figure 4, presenting the surface of targets as revealed by SE-SEM.

### 3.2. Morphological, Structural and Transport Properties of Thin Films

**Figure 5:**
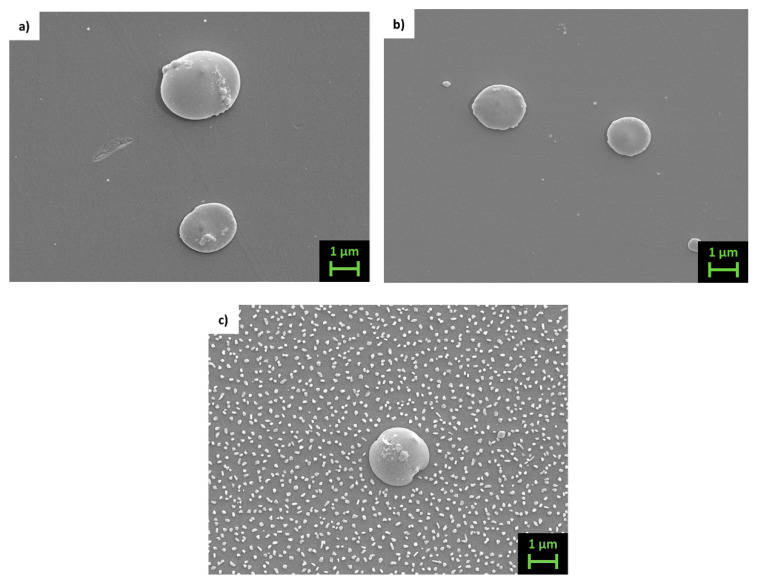
Top view LED-SEM images of samples (**a**) Fe63_RT, (**b**) Fe63_ann and (**c**) Fe63_423.

**Figure 6:**
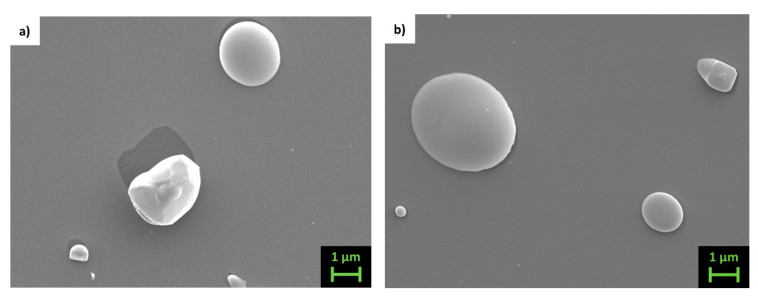

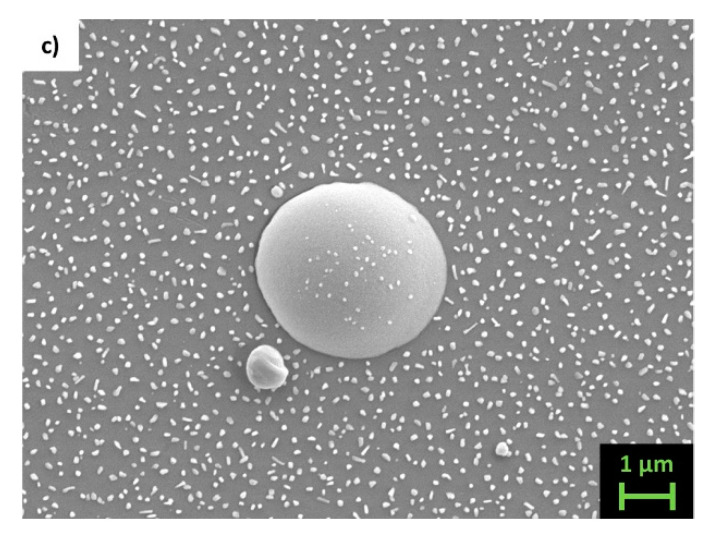
Top view LED-SEM images of samples (**a**) Fe70_RT, (**b**) Fe70_ann and (**c**) Fe70_423.

The morphology of thin films is shown in Figure 5 and Figure 6, which present LED-SEM photos of the films’ surfaces.

**Figure 7:**
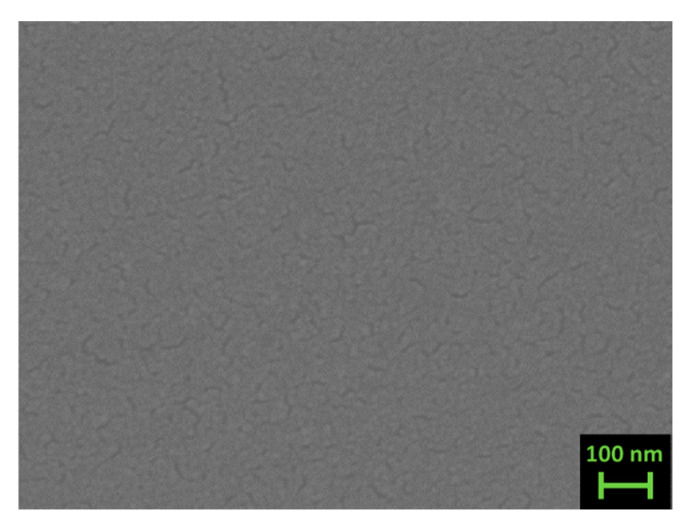
Top view high magnification LED-SEM images of sample Fe70_ann.

Films deposited at room and at high temperature present a large difference: while the former (Figure 5a,b and Figure 6a,b) show a very smooth texture, the latter (Figure 5c and Figure 6c) present uniformly distributed nanosized grains appearing on the films’ surfaces. Under a brief analysis with the EDS, they appear richer in Sb compared to the film. As can be inferred from the high magnification image taken on the sample Fe70_ann (se Figure 7), the typical grain size of these films is around 20 nm. Analogous morphology can be observed on other samples of both series. On the surface of all films, the presence of drops with sizes ranging from 1 to 2 μm is noticeable, typical of films deposited via PLD [46]. In order to reduce this inconvenience as much as possible, a study regarding the dependence of the morphology from the laser energy density is required in the future.

**Figure 8:**
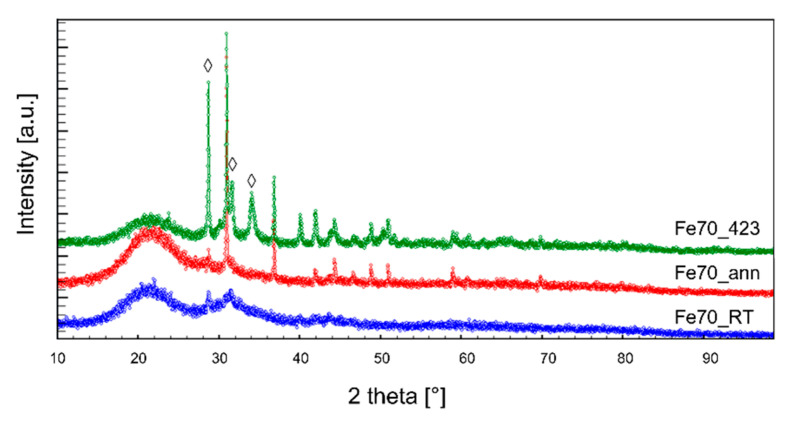
X-ray diffraction patterns of films belonging to the Fe70 series (◊ refers to the secondary phases Sm and SmSb_2_).

The X-ray diffraction (XRD) patterns collected on the three films belonging to the Fe70 series are presented in Figure 8; analogous behaviors are shown by the Fe63 series. Both samples as-deposited at room temperature (Fe63_RT and Fe70_RT) are shown to be mostly amorphous. After undergoing the annealing process, both films (Fe63_ann and Fe70_ann) exhibit the peaks corresponding to the skutterudite structure, indicating the formation of a crystalline structure. It has to be noticed that, at variance with bulk samples, annealed films present only the desired skutterudite phase, and not additional ones. On the contrary, samples deposited at 423 K (Fe63_423 and Fe70_423) show both skutterudite and undesired additional phases, such as Fe-Ni antimonides or residual-free Sb, similarly to bulk samples.

**Figure 9:**
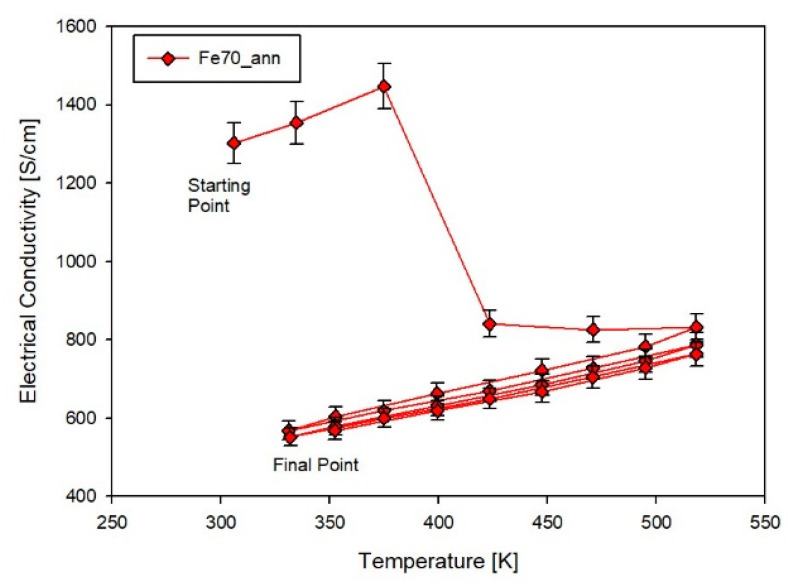
Electrical conductivity measurement cycles performed on sample Fe70_ann. Lines are guides to the eye.

The importance of the annealing process is illustrated by the electrical conductivity measurements cycling. As depicted in Figure 9, representing the σ measurement of sample Fe70_ann, it is clearly visible that during the first heating cycle up to 523 K, the *σ* drops suddenly around 325-425 K to a lower σ range, attesting to a remaining instability of the annealed films during the measurement. However, after the first heating cycle, the *σ* became perfectly reproducible; all samples follow this trend. It is highly likely that the first temperature cycle acts as a further annealing process, which promotes the obtainment of a higher crystallinity degree, even if the appearance of new phases was not observed. As a consequence, only results deriving from annealed films will be considered hereinafter.

**Figure 10:**
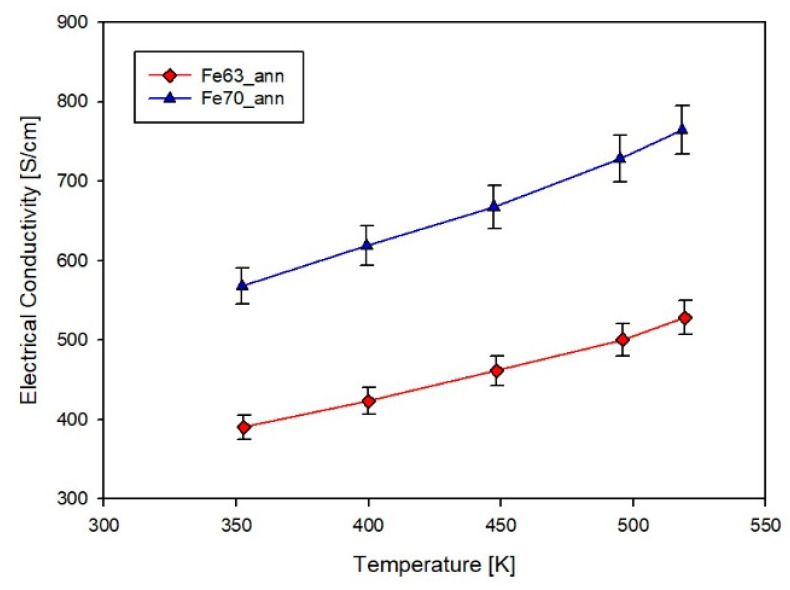
Trend of the last cycle of the electrical conductivity as a function of temperature for samples Fe63_ann and Fe70_ann.

Data reported in Figure 10, showing the trend of σ vs. temperature for both compositions of annealed films, confirm the semiconducting nature of the samples and the substantial closeness of their electrical conductivity values. A comparison with data of the (Sm,Gd)*_y_*(Fe*_x_*Ni_1−*x*_)_4_Sb_12_ skutterudite [27] indicates that the present σ values are not far from the ones of dense samples with *x* = 0.8, while they are significantly higher than those of Sm-filled bulk samples [47].

**Figure 11:**
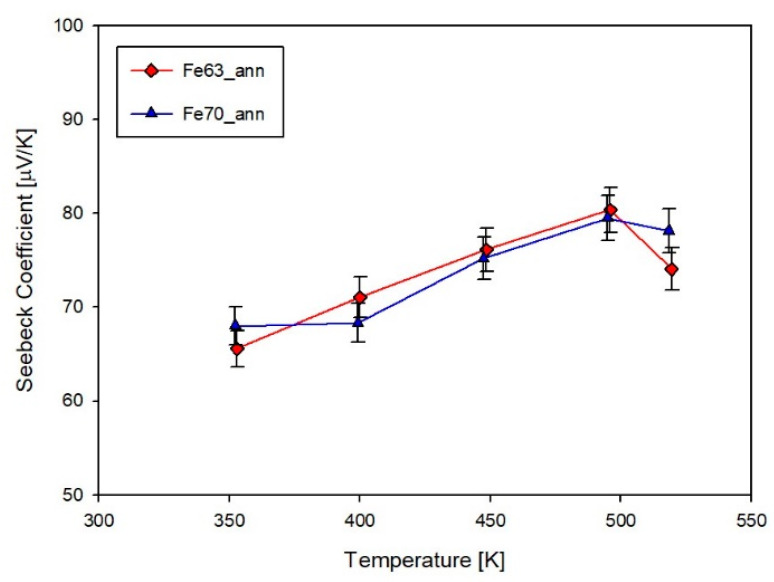
Trend of the Seebeck coefficient as a function of temperature for samples Fe63_ann and Fe70_ann.

The Seebeck coefficient S is presented in Figure 11 as a function of temperature for both annealed films. The two compositions present very similar data and result to be *p*-conducting, with values remarkably lower than the ones of (Sm,Gd)-filled compositions [27] and of Sm-filled bulk samples [47]. In comparison with thin films, the Sm-filled skutterudite bulks present larger sizes of grains (72–307 nm) and lower density (82–97%), depending on composition and on the applied sintering pressure, according with the calculations reported in [39]. The larger size of the grains and relatively lower density of the bulks with respect to films could be responsible for a higher electron scattering, which conversely yields higher values of S. As a general remark, it can be observed that both samples exhibit a weak dependence on temperature. As already observed in the two aforementioned cases, even in the present one the trend of S vs. T shows a maximum at ~500 K, as the measurement extends over a sufficiently broad temperature range.

**Figure 12:**
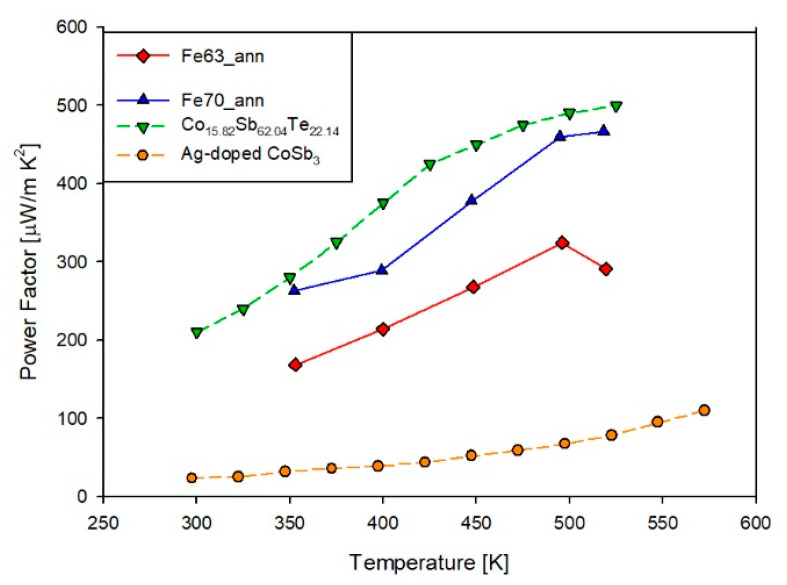
Trend of the power factor as a function of temperature for samples Fe63_ann and Fe70_ann compared to other samples in literature, namely Co_15.82_Sb_62.04_Te_22.14_ [37] and Ag-doped CoSb_3_ [48].

Making use of the measured σ and S values, the power factor ($PF=\sigma S^{2}$) has been estimated for both compositions, as shown in Figure 12. As a consequence of the aforementioned maximum in S, even in the trend of PF a maximum occurs roughly at the same temperature as in S. Regarding the absolute values of the two observed maxima, they are 323 μW/m·K^2^ and 466 μW/m·K^2^ for samples Fe63_ann and Fe70_ann, respectively, which are significantly lower than the corresponding data of the (Sm,Gd)-filled skutterudite [27]. The reason behind this behavior can be found in the cited lower value of the Seebeck coefficient with respect to the dense doubly doped system [27].

For sake of comparison, the power factor of our films is compared with other skutterudite thin films reported in literature. It can be observed that our data are comparable with the ones of Co_15.82_Sb_62.04_Te_22.14_ [37] and significantly higher than those of Ag-doped CoSb_3_ [48].

A comparison with thermal conductivity data of (Sm,Gd)-doubly doped [27], even if limited to room temperature data, shows interesting implications. Values of k of films, reported in Table 2, are very close to the ones of the doubly doped sample with *x* = 0.50 (k = 2.16 W/m·K) and only slightly higher than the one of the sample with *x* = 0.80 (k = 1.59 W/m·K), where the amount of filling ions, and hence of scattering centers, is noticeably higher. This result is very encouraging, as it suggests that the presence of interfaces acts similarly to the introduction of two different filling ions in terms of the creation of scattering centers. It can therefore be hypothesized that a better tailoring of the film composition, for instance by introducing more than one filler, or by partly substituting Sb for a proper atom, can lead toward a further reduction of thermal conductivity and an enhancement of ZT.

## 4. Conclusions

In the framework of the optimization of the thermoelectric properties of Sb-based filled skutterudites, thin films of two compositions belonging to the Sm*_y_*(Fe*_x_*Ni_1−*x*_)_4_Sb_12_ system were deposited by PLD. The system was chosen because of the promising thermoelectric performance exhibited by the corresponding bulk samples. The cited properties are expected to be improved by the introduction of further scattering centers represented by the low dimensionality and the presence of interfaces, which are typical features of films.

Depositions were performed both at room and at high (423 K) temperature; moreover, the former samples were annealed at 423 K. X-ray diffraction suggests the highest phase purity in annealed samples; accordingly, transport property values become stable only after three cycles up to 523 K, thus highlighting the importance of the annealing process. Regarding the transport properties of annealed films, electrical conductivity assumes values comparable to the ones of bulk samples, while the Seebeck coefficient is shown to be far lower, which determines lower values of the power factor. Room temperature thermal conductivity is similar in Sm-doped films and in (Gd,Sm)-doped bulk samples, thus suggesting that the presence of interfaces in films acts similarly to the introduction of different filler ions in bulk samples. The annealing process also causes the reduction of both carriers’ concentration and their mobility.

## Figures and Tables

**Table 1:** Experimental conditions of the thin film depositions.

Sample	Deposition Temperature [K]	Deposition Time [min]	Target–Substrate Distance [mm]	Annealing [min-K]
Fe63_RT	293	60	35	-
Fe63_423	423	60	35	-
Fe63_ann	293	60	35	60–423
Fe70_RT	293	60	35	-
Fe70_423	423	60	35	-
Fe70_ann	293	60	35	60–423

**Table 2:** Thickness, RT and high-T Seebeck coefficient and thermal conductivity of samples Fe63_RT, Fe63_ann, Fe70_RT and Fe70_ann.

Sample	Thickness [nm]	S @ RT [μV/K]	S @ 523K [μV/K]	k [W/m·K]
Fe63_RT	490	71.6	72.7	-
Fe63_ann	300	65.6	74.1	1.91
Fe70_RT	520	1.30	3.68	-
Fe70_ann	200	68.0	78.2	2.21

**Figure A1:**
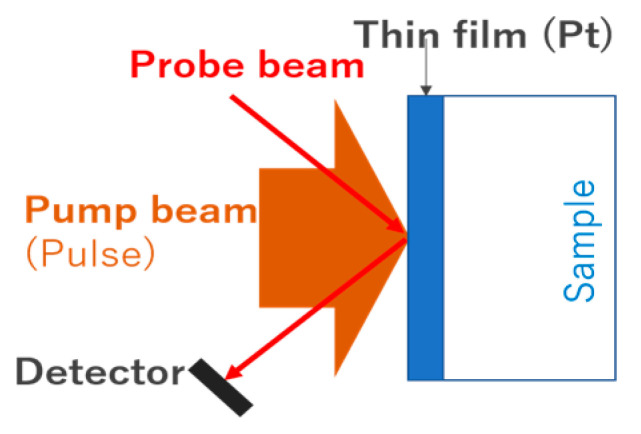
Diagram of thermo-reflectance measurement with FF configuration.

**Figure A2:**
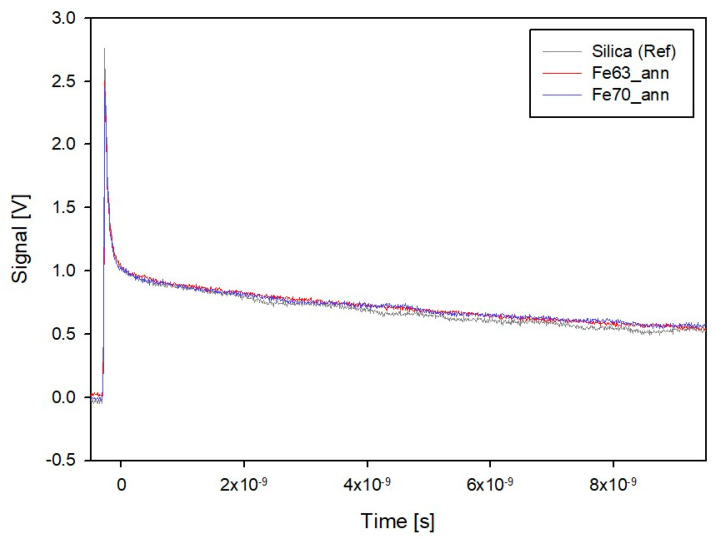
Thermo-reflectance signals of skutterudite samples.

## Data Availability

The data presented in this research study are available in this article.

## Appendix A

The picosecond time-domain thermoreflectance (TD-TR) technique using a customized focused thermal analysis system based on PicoTR (PicoTherm) was utilized to measure thermal conductivity of the samples in the cross-plane direction. For the measurements, a 100 nm layer of Pt was sputtered on the surfaces of the thin films. front-heat front-detect (FF) configuration was used (Figure A1).

To perform measurements, a 100 nm layer of Pt was sputtered on the surfaces of thin films. The front-heat front-detect (FF) configuration was employed, making use of the mirror-image method [49]. In the framework of this technique, the temperature history of the FF configuration can be explained as follows:

(A1) $$T_{f}\left(t\right)=\frac{1}{b_{f}\sqrt{\pi t}}\left(1+2\overset{\infty }{\underset{n=1}{\sum }}\gamma^{n}\exp\left({-n}^{2}\frac{\tau_{f}}{t}\right)\right)$$

(A2) $$\tau_{f\;}=\frac{d_{f}{}^{2}}{\alpha_{f}}={\left(\frac{C_{f}}{b_{f}}\right)}^{2}$$

(A3) $$\gamma =\frac{b_{f}{-b}_{s}}{b_{f}{+b}_{s}}$$

where $\tau_{f}$ is a parameter called heat diffusion time (in Pt layer), γ is a dimensionless parameter, $b_{f}$ is the thermal effusivity of Pt layer, $b_{s}$ is the thermal effusivity of the sample, $d_{f}$ is the thickness of the Pt layer and $\alpha_{f}$ is the thermal diffusivity of the Pt layer.

We already know the value of $C_{f}$, thus we can determine the thermal effusivity of the sample $b_{s}$. We can also determine thermal conductivity of the samples $\kappa_{s}$ by using following equation

(A4) $$\kappa_{s}=\frac{b_{s}{}^{2}}{c_{s}\rho_{s}}$$

where $c_{s}$ and $\rho_{s}$ are the specific heat capacity and density of the sample, respectively.

Table A1 shows the thermal properties at room temperature of annealed films; Figure A2 shows the thermo-reflectance signals obtained from the same samples and from reference substrates. For our samples we considered $c_{s}$ = 220 J/kg∙K and $\rho_{s}$ = 7360 kg/m^3^.

**Table A1:** Thermal properties of samples Fe63_ann and Fe70_ann.

Sample	Heat Diffusion Time $\tau_{f}\;[s]$	γ	Thermal Effusivity $b_{s}{\;[J/(s}^{0.5}m^{2}K)]$	Thermal Conductivity $\kappa_{s}\;[W/(m\cdot K)]$
Fe63_ann	55.5 × 10^−10^	0.747	1757	1.91
Fe70_ann	6.21 × 10^−10^	0.717	1890	2.21
